# Coping with stress produces differential systemic and brain corticosterone patterns in female rats

**DOI:** 10.3389/fnbeh.2026.1760267

**Published:** 2026-08-06

**Authors:** Emily S. Levy, Matthew G. Frank, Steven F. Maier, Michael V. Baratta

**Affiliations:** 1 Department of Psychology and Neuroscience, University of Colorado Boulder, Boulder, CO, United States; 2 Department of Integrative Physiology, University of Colorado Boulder, Boulder, CO, United States

**Keywords:** behavioral control, corticosterone, hypothalamic–pituitary–adrenal axis, prefrontal cortex, sex, stress, striatum

## Abstract

Coping factors, such as perceived or actual behavioral control over some aspect of an adverse event, are associated with resilience and are often argued to help optimize or even dampen glucocorticoid responses to stressors. Interestingly, when coping responses (i.e., stressor controllability) are experimentally manipulated in animals, controllable and uncontrollable stressors produce identical peripheral glucocorticoid responses despite eliciting markedly different behavioral outcomes. However, these studies have been conducted exclusively in male rats. In the present study, we investigated corticosterone (CORT) levels in female rats using the same controllability parameters that have been shown to produce differences in behavioral outcomes. Replicating previous studies, both controllable [escapable shock (ES)] and uncontrollable [inescapable shock (IS)] stress produced equivalent elevations in CORT levels in male rats. In contrast, in female rats, IS produced a greater increase in CORT levels than ES. We further assessed in the same animals whether initial controllability alters the CORT response to a future challenge. In male rats, but not female rats, prior IS (Time 1) sensitized the CORT response to subsequent IS (Time 2), an effect blocked by prior ES. Finally, in a separate cohort, the female controllability difference in the acute plasma CORT response was not observed in medial prefrontal cortex or striatal regions. Prefrontal expression of 11β-hydroxysteroid dehydrogenase type 1, an enzyme that regulates local concentrations of CORT, was increased in female rats exposed to ES relative to those exposed to IS. These results support the growing evidence that stress-induced differences in peripheral CORT are not necessarily reflected in brain structures that support behavioral outcomes, potentially due to regulatory mechanisms at the local tissue level.

## Introduction

Adverse life events can predispose individuals to mood disorders and other negative health outcomes that are often comorbid with these disorders (Gradus, 2017; Tian et al., 2022). However, there are substantial individual differences in responses to these aversive events, and the psychological and cognitive aspects of stressor exposure are thought to be critical determinants of these outcomes (Troy et al., 2023). One such factor, stressor controllability, is among the few psychological dimensions that can be experimentally manipulated in animals, allowing the underlying neural mechanisms to be examined in detail (Maier and Seligman, 2016). In one version of the stressor controllability task, two groups of animals (typically male rats) receive a physically identical series of tailshock stressors, but only one group has control over the termination of each trial [escapable stress (ES)], whereas the yoked group does not [inescapable stress (IS)]. IS produces numerous behavioral outcomes (e.g., impaired shuttlebox escape learning, exaggerated freezing, neophobia, and enhanced morphine-conditioned place preference), whereas physically identical ES produces none of these effects relative to non-shocked controls [home cage (HC)]. Additionally, an initial experience of ES, but not IS, leads to enduring changes that attenuate the effects of future uncontrollable adverse events (Williams and Maier, 1977; Baratta et al., 2023). Both forms of ES-mediated protection require activation of the medial prefrontal cortex (PFC) and are sex-dependent, occurring in male, but not female, rats (Fallon et al., 2020; McNulty et al., 2023).

Glucocorticoids are of interest in the context of controllability phenomena because they can coordinate responses to ongoing and pending adversity, with individual variability in responsiveness linked to differential outcomes (De Kloet et al., 2005; Caulfield and Cavigelli, 2020). Stress exposure efficiently activates the hypothalamic–pituitary–adrenal (HPA) axis and elicits the release of corticosterone (CORT) into the circulation, where it can enter the brain and influence behavior through both non-genomic (rapid) and genomic (delayed) actions (Dallman, 2005; Myers et al., 2014). These actions are under proximal control by the HPA axis and are subject to considerable modification by factors such as prior history and features of the stress context (Grissom et al., 2007; Hennessy et al., 2009; Lyons et al., 2010; Herman, 2013). Yet, despite its stress-buffering behavioral and neurochemical effects, behavioral control does not reduce peripheral CORT levels relative to uncontrollable stress, either immediately following the shock session (Maier et al., 1986; Helmreich et al., 1999; Frank et al., 2025) or during subsequent behavioral testing (Maier et al., 1986).

Moreover, this pattern extends to other components of the HPA system. Tailshock increases peptide mRNA expression within hypothalamic paraventricular neurons that provide a stimulatory drive on pituitary–adrenal activity. Both ES and IS produce similar levels of corticotropin-releasing hormone, neurotensin, enkephalin, and arginine vasopressin (Helmreich et al., 1999). Similarly, neither the magnitude nor the decay rate of plasma adrenocorticotropic hormone levels is altered by the presence of control (Maier et al., 1986). Certainly, experimental conditions exist in which controllable and uncontrollable stressors produce different HPA responses (Dess et al., 1983; Swenson and Vogel, 1983; Prince and Anisman, 1990), but the exact ES/IS conditions that produce well-studied and frequently replicated behavioral and neurochemical differences do not produce HPA differences.

However, a recent report by Frank et al. (2025) demonstrated a dissociation between peripheral and brain CORT levels in male rats. ES selectively attenuated PFC CORT levels and 11β-hydroxysteroid dehydrogenase type 1 (11β-HSD1), the steroidogenic enzyme that converts inert 11-dehydrocorticosterone (11-DHC) to active CORT (Chapman et al., 2013). These ES effects were absent in other brain regions, indicating a region-specific pattern of tissue CORT regulation that is not reflected in the blood.

Whether controllability impacts either central or peripheral glucocorticoid levels in female rats remains largely unexplored. This is an important issue because controllability affects both behavior and neurochemistry quite differently in male and female rats (Fallon et al., 2020; McNulty et al., 2023). Thus, the present study addressed three separate issues: First, we tested whether the CORT response to an initial session of ES vs. an equal amount of IS differs in female rats. The shock parameters and procedures used were identical to those that elicit the differential behavioral sequelae in male rats. Second, we examined whether prior experience with controllability alters CORT responses to subsequent IS exposure 1 week later. It is possible that ES and IS modify the HPA response to future adverse events (e.g., sensitization or habituation), even if their initial responses are equivalent. Finally, in female rats, we assessed whether stress-induced increases in brain CORT levels are modulated by behavioral control.

## Materials and methods

### Animals

Female and male adult Sprague–Dawley rats (Envigo, Indianapolis, IN) were provided with Teklad Global Diet 2918 (Envigo) and water *ad libitum* and were maintained on a 12-h light/dark cycle (lights on at 0700). Rats were pair-housed and allowed to acclimate to colony conditions for at least 1 week before experimentation. All behavioral procedures were carried out between 0900 and 1300 h. Experiments were approved by the University of Colorado Boulder Institutional Animal Care and Use Committee and were conducted in accordance with the National Institutes of Health *Guide for the Care and Use of Laboratory Animals*.

### Wheel-turn escape/yoked IS procedure

The procedure was identical to that used in previous studies assessing the effects of controllability on stressor outcomes in rats (McNulty et al., 2023). Rats were tested in triads. One subject of each triad received ES, a second received yoked IS, and a third remained undisturbed in the colony (home cage, HC). Each ES and IS rat was placed in a small individual Plexiglass box with a wheel mounted in the front. The tail was secured to a Plexiglass rod extending from the back of the box and fitted with two copper electrodes and electrode paste. The stress session consisted of 100 tailshock trials (1.0 mA) presented on a 60-s variable interval (VI) schedule. Initially, the shock could be terminated by a quarter turn of the wheel by the ES rat. When trials were completed in less than 5 s, the response requirement increased by one-quarter turn, up to a maximum of four full turns. The response requirement was reduced if the trial was not completed within 5 s. Failure of the ES rat to respond within 30 s terminated the trial and reset the wheel-turn requirement to the initial one-quarter turn. This experimental arrangement ensured that (i) ES subjects maintained efficient escape behavior and (ii) shock duration on any given trial remained within the 3–5 s range. The yoked IS subject simply received the same shock duration on each trial that was received by its ES partner.

### Inescapable shock procedure

In Experiment 1, all rats received a second stress session 1 week later, which involved fixed-duration IS in a different apparatus (a Plexiglas tube, 17.5 cm in length and 6.0 cm in diameter). Rats received 100 inescapable tailshocks (5 s in duration at 1.0 mA each) delivered on a VI schedule ranging from 30 to 90 s (with an average interval of 60 s).

### Tissue collection

All tissue collection procedures were conducted in a separate room isolated from both the colony and the stress procedure. In Experiment 1 (*n* = 8 animals per group), serial measures of plasma CORT were obtained via rapid lateral tail vein incision at three time points on the day of stress exposure: before the stress session (baseline), immediately after the end of the stress session (0 h), and 4 h later. The same time points were used for blood samples collected 1 week later for the second stress session. Blood was directly collected into heparin-treated 1.5 mL Eppendorf microcentrifuge tubes. Samples were immediately centrifuged (for 10 min at 4,000 × *g* and 4 °C), and the resulting supernatant was pipetted into microcentrifuge tubes and stored at −70 °C.

In Experiment 2 (*n* = 5–8 animals per group), immediately after the end of the stress session, rats were anesthetized with isoflurane and transcardially perfused with ice-cold 0.9% saline to remove blood from the brain vasculature. Whole brains were dissected, flash-frozen in isopentane, and stored at −70 °C. Brains were sectioned on a Leica cryostat at −20 °C until the region of interest was reached. Bilateral micropunches were extracted from brain regions using a brain punch tool (1 mm diameter x 1 mm depth) and stored at −70 °C until analysis. Targeted regions included the PFC, dorsomedial striatum (DMS), dorsolateral striatum (DLS), and ventral tegmental area (VTA), all of which have been implicated in mediating the differential behavioral outcomes between male and female ES subjects (Baratta et al., 2023; McNulty et al., 2023).

### Enzyme-linked immunosorbent assay (ELISA)

Tissue micropunches were sonicated using a tissue extraction reagent (Thermo Fisher Scientific, Waltham, MA, USA, Cat# FNN0071) supplemented with a protease inhibitor cocktail (MilliporeSigma, Burlington, MA, USA, Cat# P2714). Homogenates were centrifuged (for 10 min at 14,000 × *g* and 4 °C), and the supernatants were collected and stored at −70 °C. Total protein concentration was quantified using a Bradford assay. Blood and brain CORT concentrations were determined using a competitive immunoassay (Enzo Life Sciences, Farmingdale, NY, USA, Cat# ADI-900-097, RRID: AB_2307314) according to the manufacturer’s protocol.

### Western blotting

Tissue homogenates (10 μg total protein) were diluted in lithium dodecyl sulfate (1x) sample buffer (Thermo Fisher Scientific, Cat# NP0007) and dithiothreitol (50 mM, Thermo Fisher Scientific, Cat# NP0004), heated to 70 °C for 10 min, and loaded onto a standard polyacrylamide 4–12% Bis-Tris gel (Thermo Fisher Scientific, Cat# NP0323BOX). SDS-PAGE was performed in NuPAGE MES SDS Running Buffer (Thermo Fisher Scientific, Cat# NP0002) at 175 V for approximately 45 min. Proteins were transferred to a nitrocellulose membrane using an iBlot dry transfer system (Thermo Fisher Scientific, Cat# IB001). The membrane was then blocked with Odyssey Blocking Buffer (LI-COR, Lincoln, NE, Cat# 927-4000) for 1 h and incubated overnight at 4 °C in Odyssey Blocking Buffer containing primary antibodies: rabbit polyclonal to 11β-HSD1 (1:1,000, Thermo Fisher Scientific, Cat# PA5-21586, RRID: AB_11153989) and mouse monoclonal to β-actin (1:100,000, Sigma-Aldrich, St. Louis, MO, Cat# A5316, RRID: AB_476743). Membranes were washed four times in 1× PBS + 0.1% Tween and then incubated for 1 h at room temperature in Odyssey Blocking Buffer containing goat anti-rabbit (LI-COR, Cat# 925-32211, RRID: AB_2651127) and goat anti-mouse (LI-COR, Cat# 925-32210, RRID: AB_2687825) IRDye 800CW secondary antibodies (1:10,000). Membranes were washed again four times in 1x PBS + 0.1% Tween. Protein expression was quantified using the Odyssey XF Imaging System (LI-COR, RRID: SCR_022510) and normalized to the housekeeping protein β-actin.

### Data analysis

Data were analyzed using Prism 10.6.0 (GraphPad, Boston, MA, RRID: SCR_002798). CORT analyses were designed to assess, within each sex, whether behavioral control modulates stress-induced CORT responses. Secondary analyses included testing for a sex × stress treatment interaction at the 0 h blood collection time point for both stress sessions. CORT and 11β-HSD1 protein datasets were first assessed for normality; when normality was not met, a non-parametric test (the Kruskal–Wallis test) was used. Wheel-turn escape performance (response requirement and latency) and plasma CORT levels were analyzed using a mixed-model repeated-measures analysis of variance (ANOVA). Depending on the analysis, either sex or stress treatment (ES, IS, and HC) was included as the between-subjects factor, and trial block or blood collection time point served as the within-subject factor. Brain CORT and 11β-HSD1 protein levels were analyzed with a one-way ANOVA. In the event of significant main effects and interactions, *post-hoc* comparisons were performed using the Benjamini false discovery rate (FDR) procedure to identify the locus of significant differences. Statistical significance was set at *α* = 0.05. The results are presented as mean ± SEM, and sample sizes are provided in the figure legends.

## Results

**Figure 1:**
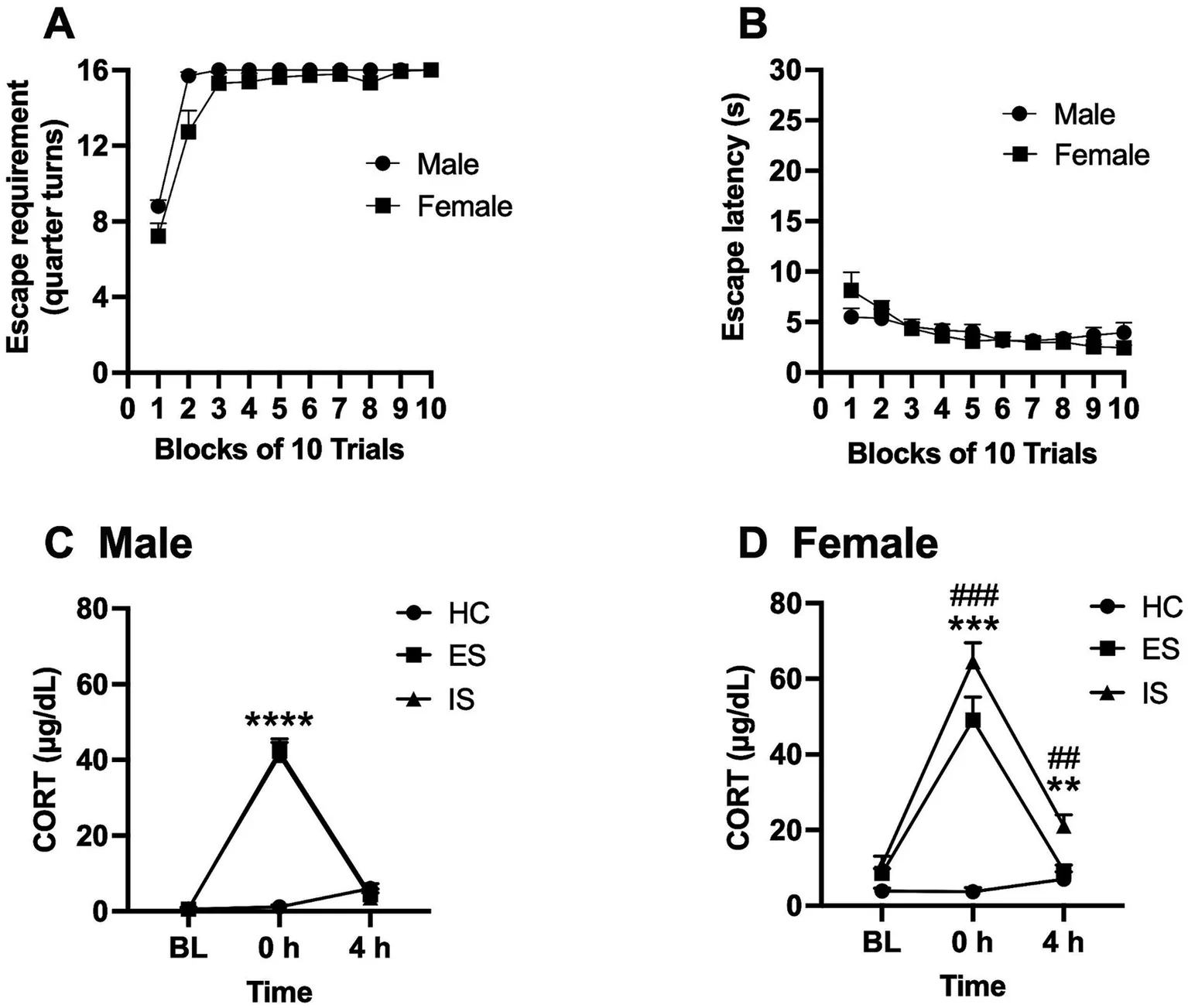
Male and female plasma corticosterone (CORT) concentration (μg/dL) following escapable stress (ES), inescapable stress (IS), and home cage control (HC). Efficiency of wheel-turn escape behavior during exposure to controllable tailshock showing the **(A)** number of quarter-turns of the wheel attained as the escape requirement on each trial and **(B)** escape latencies (s) for ES subjects in all experiments. *N* = 8 males, 15 females. **(C)** Male and **(D)** female serial measures of plasma CORT were obtained before the stress session (baseline, BL), immediately after the end of the stress session (0 h), and 4 h later. *N* = 8 animals per group. FDR-adjusted *post-hoc* comparisons; vs. HC **p < 0.01, ***p < 0.001, ****p < 0.0001, ES vs. IS ^##^*p* < 0.01, ^###^*p* < 0.001.

Across both experiments, male and female ES subjects learned the controlling wheel-turn response with proficiency equivalent to that reported in previous studies (Baratta et al., 2019). The quality of escape performance, including both the response requirement attained across trials (number of quarter wheel turns to terminate the shock) and the time taken to terminate each tailshock (escape latency), is shown in Figures 1A,B, respectively. For both measures, a repeated-measures ANOVA indicated neither a main effect of sex (response requirement: *F*_(1,21)_ = 2.83; latency: *F*_(1,21)_ = 0.03, *p’s* > 0.05) nor an interaction of sex and time (response requirement: *F*_(9,189)_ = 1.92; latency: *F*_(9,189)_ = 1.46, *p’s* > 0.05).

### Experiment 1: effect of stressor controllability on the plasma CORT response in male and female rats to acute and subsequent tailshock

Male plasma CORT levels following ES, yoked-IS, and HC conditions are shown in Figure 1C. Tailshock produced a pronounced increase in plasma CORT immediately after the 100-trial session, which returned to HC levels at 4 h. Consistent with previous studies (Maier et al., 1986; Helmreich et al., 1999), male ES and IS subjects did not differ at any time point. Female rats exhibited a different pattern (Figure 1D). Both ES and IS produced elevated CORT levels; however, IS resulted in significantly higher CORT concentrations at both post-stress time points.

Repeated-measures ANOVA revealed main effects of time (*F*_(2,42)_ = 207.2, *p* < 0.0001), treatment (*F*_(2,21)_ = 44.84, *p* < 0.0001), and a significant interaction between time and treatment (*F*_(4,42)_ = 59.82, *p* < 0.0001) on male CORT levels. At 0 h post-stress, both male ES (42.30 μg/dL) and IS (41.20 μg/dL) rats exhibited elevated CORT levels relative to HC (1.17 μg/dL; *p*s < 0.0001), but did not differ significantly from one another (*p* > 0.05). For female rats, stressor exposure produced a significant main effect of time (*F*_(2,42)_ = 91.50, *p* < 0.001), treatment (*F*_(2,21)_ = 59.43, *p* < 0.001), and a significant interaction between time and treatment (*F*_(4,42)_ = 26.57, *p* < 0.001). Immediately after the stress session, female ES (49.13 μg/dL) and IS (64.48 μg/dL) CORT concentrations were elevated compared to HC (3.69 μg/dL; *p*s < 0.001). Unlike male rats, female IS rats exhibited higher CORT levels than ES rats at both 0 h (*p* < 0.001) and 4 h time points (*p* < 0.01). A two-way ANOVA (sex × stress treatment) at 0 h revealed a significant interaction (*F*_(2,42)_ = 4.18, *p* < 0.05), with ES and IS groups differing in female rats (*p* < 0.01), but not in male rats (*p* > 0.05).

**Figure 2:**
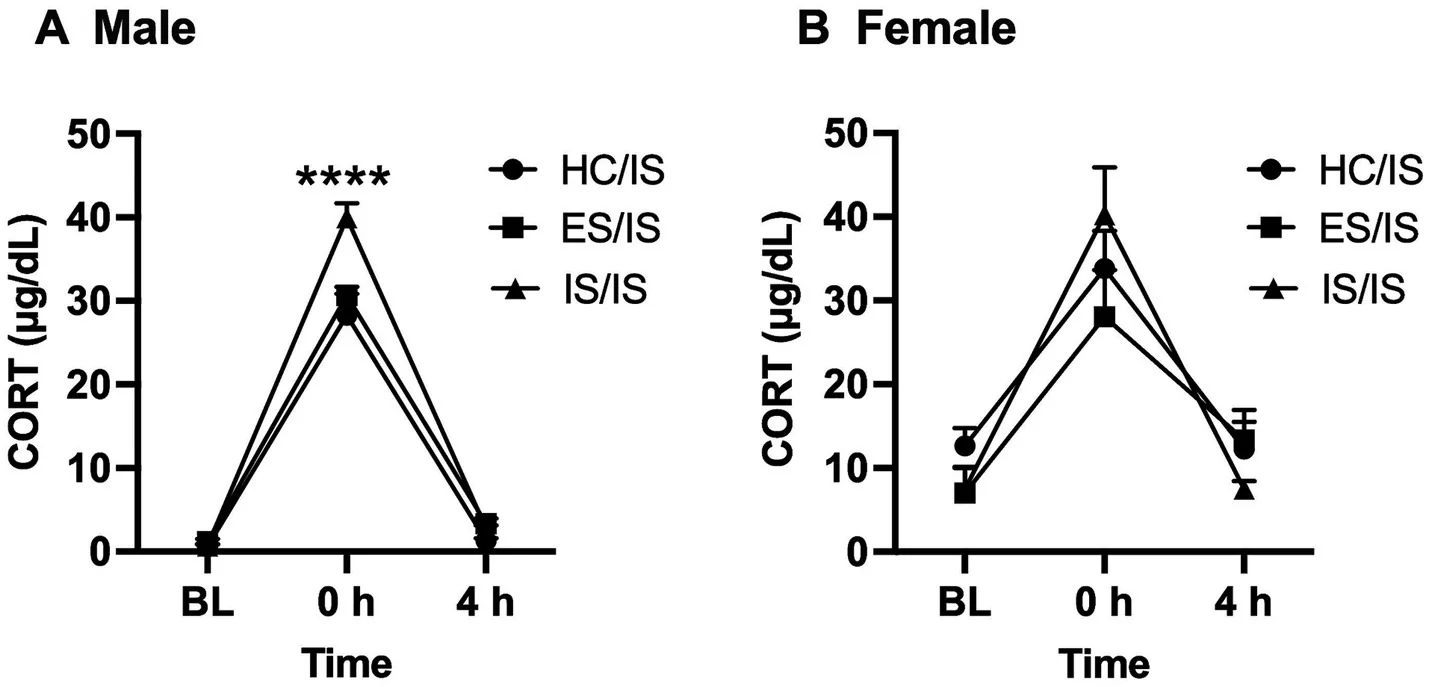
Male and female plasma corticosterone (CORT) concentration (μg/dL) following inescapable stress (IS) from groups of rats that received escapable stress (ES), IS, and home cage control (HC) 1 week prior. **(A)** Male and **(B)** female serial measures of plasma CORT were obtained before the second stress session (baseline, BL), immediately after the end of the second stress session (0 h), and 4 h later. *N* = 8 animals per group. FDR-adjusted *post-hoc* comparisons; IS vs. ES and HC ****p < 0.0001.

Because acute stressors often alter HPA responsiveness to subsequent stress exposure (Martí et al., 2001; Campeau et al., 2002; Belda et al., 2015), we examined whether initial ES and IS (Time 1) influenced CORT secretion in response to later IS (Time 2). Thus, 1 week later, all subjects underwent a second stress session, consisting of 100 IS trials, each delivered for a fixed duration of 5 s (1.0 mA), in a distinctly different context (a Plexiglass tube). In male rats, prior IS, but not ES, produced a sensitized CORT response to subsequent IS (Figure 2A). This IS-induced sensitization was absent in female rats (Figure 2B).

For male rats, main effects of time (*F*_(2,42)_ = 732.4, *p* < 0.0001), treatment (*F*_(2,21)_ = 11.45, *p* < 0.001), and the interaction between time and treatment (*F*_(4,42)_ = 9.11, *p* < 0.0001) were all significant. Subsequent *post-hoc* comparisons revealed that rats previously exposed to IS exhibited higher CORT levels (39.92 μg/dL) at the initial blood collection time point (0 h) compared to those previously exposed to ES (30.66 μg/dL) and HC rats (28.25 μg/dL; *p’s* < 0.0001), which did not differ from each other (*p* > 0.05). For female rats, there were no significant effects of treatment (*F*_(2,21)_ = 0.584, *p* > 0.05) or the interaction between time and treatment (*F*_(4,42)_ = 1.72, *p* > 0.05) on CORT levels. A significant main effect of time (*F*_(2,42)_ = 41.98, *p* < 0.001) reflected increased CORT levels in all groups (prior ES, 28.04, prior IS, 40.21; and prior HC, 33.81 μg/dL) immediately after the second stress session (0 h, *p* < 0.001), which returned to baseline levels by 4 h (*p* > 0.05). A subsequent two-way ANOVA (sex × stress treatment) failed to reveal a main effect of sex (*F*_(2,42)_ = 0.11, *p* > 0.05) or an interaction between sex and treatment at the 0 h time point (*F*_(2,42)_ = 0.54, *p* > 0.05).

### Experiment 2: effect of stressor controllability on brain CORT levels in female rats

**Figure 3:**
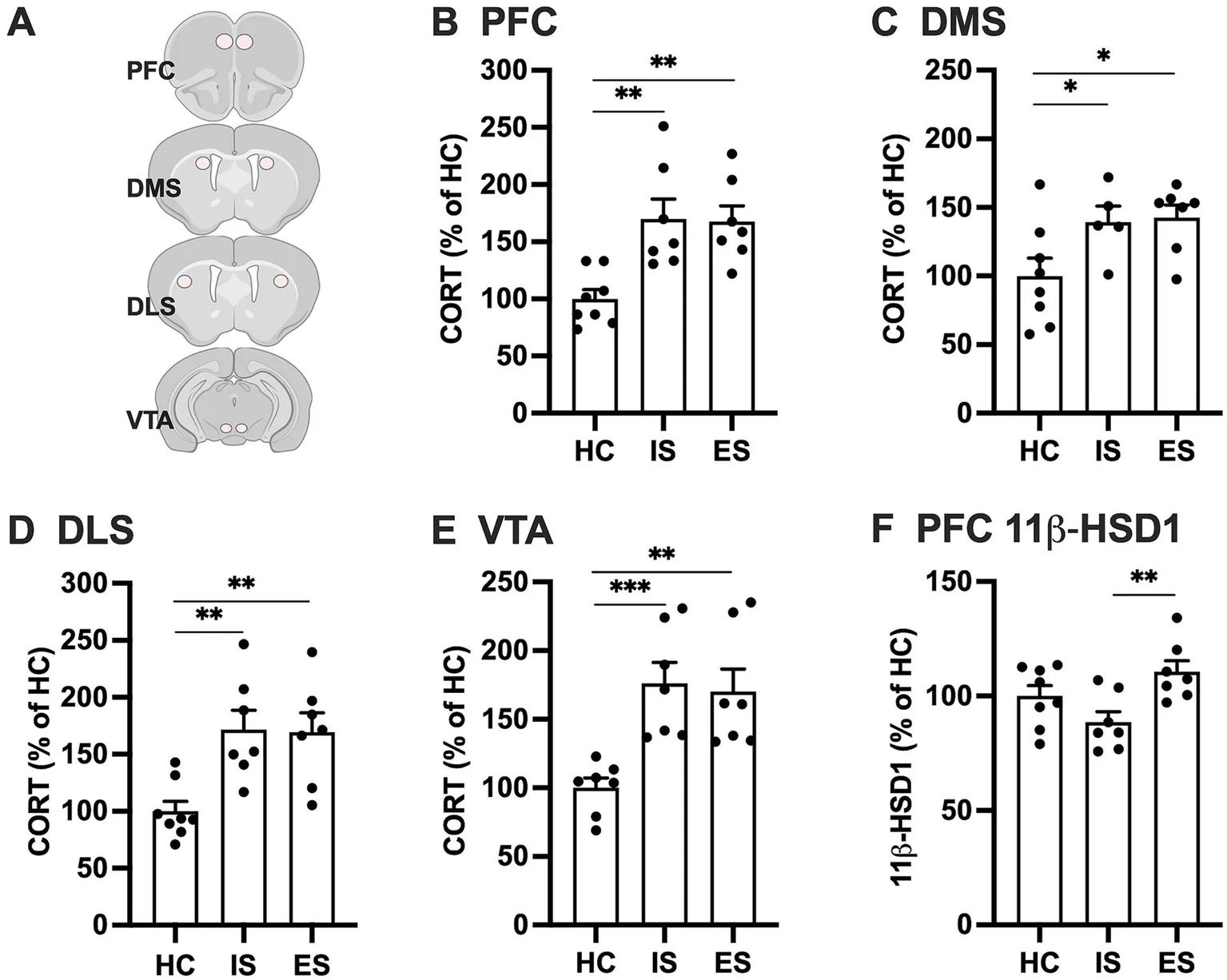
Effect of controllability on brain corticosterone (CORT) levels in female rats exposed to escapable stress (ES), inescapable stress (IS), and home cage control (HC). **(A)** Schematic illustration of brain micropunch locations. CORT levels were measured in **(B)** medial prefrontal cortex (PFC), **(C)** dorsomedial striatum (DMS), **(D)** dorsolateral striatum (DLS), and **(E)** ventral tegmental area (VTA) from brains collected immediately after the stress procedure. **(F)** Protein expression of 11β-hydroxysteroid dehydrogenase type 1 (11β-HSD1) in PFC. Data are presented as % of HC. *N* = 5–8 animals per group. FDR-adjusted *post-hoc* comparisons; *p < 0.05, **p < 0.01, ***p < 0.001. **(A)** was created with BioRender.com.

Our previous studies in male rats demonstrated that behavioral control attenuates PFC CORT levels (Frank et al., 2025). Therefore, in a separate female cohort, we examined whether the differential peripheral CORT response observed between the ES and IS female groups (Figure 1D) was also present in brain regions (Figure 3A) previously linked to the lack of stress protection in ES female rats (see the Introduction section). As shown, local CORT tissue levels were elevated following shock across the PFC (Figure 3B) and the striatal subregions examined (Figures 3C–E). However, CORT levels in the ES and IS groups were equivalent in all cases, revealing a pattern distinct from that observed in blood.

In the PFC, stressor exposure significantly increased CORT levels compared to HC (*F*_(2,19)_ = 9.40, *p* < 0.01; ES vs. HC, *p* < 0.01, IS vs. HC, *p* < 0.01). However, the ES and IS groups did not differ (*p* > 0.05). Controllability also failed to modulate shock-induced CORT levels in the striatal subregions, with only a main effect of stress on CORT reaching significance in the DMS (*F*_(2,17)_ = 4.42, *p* < 0.05), DLS (*F*_(2,19)_ = 8.41, *p* < 0.01), and VTA (H[2] = 13.51, *p* < 0.0001).

Discrepancies between peripheral and brain CORT levels can arise due to the expression of steroidogenic enzymes in the brain, including 11β-HSD1, which converts inactive 11-DHC to active CORT (Salehzadeh and Soma, 2021; Seckl, 2024). We therefore examined the effect of controllability on 11β-HSD1 protein expression in the PFC (Figure 3F). Western blot analysis revealed a main effect of treatment (*F*_(2,19)_ = 5.28, *p* < 0.05), reflecting a selective enhancement of 11β-HSD1 expression in female rats exposed to ES compared with IS (*p* < 0.001).

## Discussion

The present results provide initial evidence of sex differences in how controllability modulates the glucocorticoid response to stressors. A single session of equal duration controllable (ES) and uncontrollable (IS) tailshock led to a robust increase in plasma CORT concentrations, with male rats exhibiting equivalent levels after ES and IS, whereas female rats showed a significantly higher response to IS than ES. The pattern observed in the male rats is consistent with previous findings obtained using similar parameters to those employed here (Maier et al., 1986; Helmreich et al., 1999; Frank et al., 2025). Plasma CORT values were nearly twice those reported previously (Maier et al., 1986), most likely reflecting the greater assay sensitivity in the present study (ELISA, 27.0 pg./mL) (Bekhbat et al., 2018) compared with earlier methods (radioimmunoassay, 1.0 ng/mL). Although ES and IS produce markedly different behavioral and neurochemical effects in male rats, the collective findings do not support the expectation that these differences would be accompanied by corresponding differences in HPA activity during the initial control experience.

Although the initial HPA response did not differ, prior ES proactively blunted the CORT response to subsequent IS exposure 1 week later in a novel environment. This finding is novel and was observed only in male rats. The shock re-exposure procedure was identical to that used by Der-Avakian et al. (2005), who reported that prior IS enhances the CORT response to morphine in male rats and that the CORT response to the drug, but not to the stressor, is necessary for the IS-induced increase in morphine-conditioned place preference. IS-induced sensitization of the CORT response to subsequent HPA activation generalizes broadly (Johnson et al., 2002), but the role of controllability in shaping future CORT responses had not previously been addressed. It may be that ES leads to a long-lasting alteration in some component of the HPA axis that is expressed during subsequent HPA activation but not at the time of initial ES exposure (Daviu et al., 2020).

In contrast to male rats, behavioral control does not confer stress resilience in female rats. Female rats exposed to ES exhibit behavioral outcomes equivalent to those observed following IS—exaggerated fear, social avoidance, and impaired shuttlebox performance (Baratta et al., 2018). Furthermore, ES neither buffers against future adverse events (Baratta et al., 2019) nor facilitates dominance, as it does in male rats (Coleman et al., 2024). Despite this behavioral equivalence, during the initial stressor exposure at Time 1, ES led to an attenuated CORT response in female rats compared with IS at both 0 and 4 h post-stress. These findings reveal a dissociation between circulating CORT levels and the behavioral profile typically associated with controllability in female rats. One week later, at Time 2, prior exposure to either ES or IS did not alter the CORT response to a subsequent IS experience in a novel environment. Although the IS/IS group showed a trend toward a greater increase in CORT from baseline to its peak level than the HC/IS or ES/IS groups, overall variability in female rats may have limited the detection of CORT sensitization.

It is unclear what aspect of the ES experience enables modulation of the HPA response in female rats but not in male rats. Estrous phase can potently shape HPA responsivity and glucocorticoid output, and future studies should determine whether the effect of ES is similarly effective under all hormonal conditions. We also observed that the performance of the instrumental control response (turning a wheel to terminate shock) was virtually identical between sexes (Figure 1). Shock parameters (duration, intensity, and temporal pattern) and the number of wheel-turn responses required to terminate shock (fixed-ratio requirement) did not differ. The neural mediation of instrumental performance, however, can be supported by two distinct systems (Balleine et al., 2007). One, the action-outcome system, has an associative structure in which specific responses are sensitive to their outcomes (R-O). Devaluation of the outcome or weakening of the response–outcome contingency reduces responding. In contrast, responses governed by the habit system are elicited by contextual or environmental cues without regard to their consequences (S-R); performance is autonomous of the current value of the outcome. Action-outcome learning and performance depend, in part, on a corticostriatal circuit involving prelimbic cortex projections to the DMS, whereas reflexive, habitual responding is mediated by the DLS, which operates independently of the PFC. This distinction is important because the wheel-turning ES task in male rats engages action-outcome circuitry, while female rats rely on the habit system (Baratta et al., 2023; McNulty et al., 2023).

Thus, the dissociation in CORT levels between female and male ES rats could be explained if engaging habit-based processes reduces glucocorticoid levels. The literature is not extensive or definitive, but several studies have examined the interaction between HPA activity and instrumental performance. Stress-induced increases in CORT, which are often higher in female rats (Heck and Handa, 2019), can bias learning and performance to the habit system (Wingard and Packard, 2008; Dias-Ferreira et al., 2009; Schwabe and Wolf, 2011; Vogel et al., 2017; Gadberry et al., 2022). However, the reverse direction of this relationship, *how engaging in habit-like behaviors influences HPA activity*, has unfortunately received less attention. Some evidence suggests that constructs marked by structured, repetitive behavior (habit, stereotypy, and routine) may, under certain conditions, exert stress-reducing effects by enhancing predictability and efficiency (Valenstein, 1976; Jeremiah et al., 2025). Drug-induced stereotyped behavior (Jones et al., 1989; Mittleman et al., 1991), repeated voluntary exercise (Campeau et al., 2010; Hare et al., 2014), and prolonged training in aversively motivated instrumental behavior (Coover and Ursin, 1973; Davis et al., 1977) can attenuate HPA responses to challenge. Although speculative, female recruitment of habit circuitry during instrumental control may similarly lead to reduced circulating CORT.

The extent to which brain CORT parallels group differences in peripheral levels is largely unexamined in the context of factors that confer stressor resistance/resilience. Tissue glucocorticoid levels can be independently regulated through regeneration and metabolism (Chapman et al., 2013; Hamden et al., 2021), and local concentrations can vary across brain regions in response to challenge (Hamden et al., 2025). We previously found that ES in male rats blunts tissue CORT levels and completely blocks the stress-induced increase in 11β-HSD1 (Frank et al., 2025). These effects were specific to the PFC, suggesting that behavioral control may regulate CORT action within action-outcome circuitry rather than through systemic inhibition of HPA activity. In the present study, controllability in female rats did not alter tissue-level increases in CORT across prefrontal and striatal brain regions implicated in mediating the differential behavioral outcomes between male and female ES subjects. As tissue CORT was assessed only immediately after the stressor session, differential responses at other time points remain possible. ES in female rats also increased PFC 11β-HSD1 protein expression relative to IS. The functional implications of this increase remain unclear, but elevated 11β-HSD1 may serve to amplify local CORT availability, potentially accounting for the dissociation between tissue and plasma CORT levels. Whether regulation of glucocorticoids in corticostriatal circuitry influences how instrumental control is processed at a system level and/or how such learning shapes future responses to adversity remains to be determined through further experimentation.

## Data Availability

The raw data supporting the conclusions of this article will be made available by the authors, without undue reservation.
